# Nova Scotia Health Officers Combat Alcoholism

**Published:** 1916-09

**Authors:** 


### Abstracts and Selections.

### Nova Scotia Health Officers Combat Alcoholism.

The following resolutions adopted by medical health officers and practicing physicians in Nova Scotia will be read with interest. The first resolution was passed at the meeting of the Medical Society of Nova Scotia held in July; the second was adopted by the Conference of Medical Health Officers of Nova Scotia in September:

"That since it has been established that alcohol is not a food, in that none of its elements is incorporated into the tissues, and since the heat it produces by oxidation is over-compensated for through heat loss from the blood-vessels of the skin, and since alcohol is not required to aid any physiological process, and since by its excessive use all systems of the body are injured and the moral nature so altered as to lead to crime, this meeting desires to impress the community with the benefits to be obtained by abstinence from alcohol as a beverage, and recommends its use only under medical advice. This meeting would warn the public that many patent medicines containing large amounts of alcohol are neither foods nor stimulants as advertised."

"WHEREAS, It has been absolutely proven that alcohol has a pernicious and injurious effect on the public health of our country, in that it lowers the resistance of the individual to disease, thereby disposing to tuberculosis and other infectious diseases; and

"WHEREAS, It is one of the chief contributing factors to poverty, misery and crime: therefore,

"We, as Health Officers of the Province of Nova Scotia, place ourselves on record as opposed to its use as a beverage and strongly recommend its use only upon medicial prescription."

---

The following article by Dr. Ludvig A. Brustad of San Antonio, Texas, was published in the *American Journal of Electrotherapeutics and Radiology,* May, 1916. It is a forceful argument and an earnest plea for a better understanding, upon the part of the general practitioner, of the wonderful results that are being obtained in infantile paralysis, by the use of electrotherapy. EDITOR.

